# Bacterial Composition, Community Structure, and Diversity in *Apis nigrocincta* Gut

**DOI:** 10.1155/2020/6906921

**Published:** 2020-07-30

**Authors:** Christian Apolinaris Lombogia, Max Tulung, Jimmy Posangi, Trina Ekawati Tallei

**Affiliations:** ^1^Entomology Study Program, Postgraduate Program, Sam Ratulangi University, Manado, North Sulawesi, Indonesia; ^2^Nursing Study Program, Faculty of Nursing, De La Salle Catholic University, Manado, North Sulawesi, Indonesia; ^3^Public Health Study Program, Faculty of Public Health, Sam Ratulangi University, Manado, North Sulawesi, Indonesia; ^4^Department of Biology, Faculty of Mathematics and Natural Sciences, Sam Ratulangi University, Manado, North Sulawesi, Indonesia

## Abstract

Understanding the honeybee gut bacteria is an essential aspect as honeybees are the primary pollinators of many crops. In this study, the honeybee-associated gut bacteria were investigated by targeting the V3-V4 region of 16S rRNA genes using the Illumina MiSeq. The adult worker was captured in an urban area in a dense settlement. In total, 83,018 reads were obtained, revealing six phyla from 749 bacterial operational taxonomic units (OTUs). The gut was dominated by Proteobacteria (58% of the total reads, including Enterobacteriaceae 28.2%, *Erwinia* 6.43%, and *Klebsiella* 4.90%), Firmicutes (29% of the total reads, including *Lactococcus garvieae* 13.45%, *Lactobacillus* spp. 8.19%, and *Enterococcus* spp. 4.47%), and Actinobacteria (8% of the total reads, including *Bifidobacterium* spp. 7.96%). Many of these bacteria belong to the group of lactic acid bacteria (LAB), which was claimed to be composed of beneficial bacteria involved in maintaining a healthy host. The honeybee was identified as *Apis nigrocincta* based on an identity BLAST search of its COI region. This study is the first report on the gut microbial community structure and composition of *A. nigrocincta* from Indonesia.

## 1. Introduction

Honeybees, members of the genus *Apis*, are social insects, that are famous for their honey production. Nine species of honeybees are currently known to inhabit the world: *Apis dorsata*, *A. laboriosa*, *A. mellifera*, *A. florea*, *A. andreniformis*, *A. cerana*, *A. koschevnikovi*, *A. nigrocincta*, and *A. nuluensis*. Honeybees play indispensable roles in pollination. However, their population tends to decline in recent years [1]. This decline will cause severe problems in pollination services in both agricultural and natural settings, thus threatening the world's food supply.

Honeybees have become essential models in studying the influence of microbial communities with their hosts [2]. Various factors, among others, govern the complex microbiome community in the gut of the insects, is the flowers they visit. The insect hosts provide an environment that supports microbial growth in the gut, which benefits the hosts [3], such as synthesizing and absorbing nutrients. Several studies reveal that their microbial community may be involved in pathogen defense [4] and play significant roles in the growth, development, and environmental adaptation of the host, as well as bioprospecting [5].

It is estimated that there are approximately one billion bacterial cells in an adult workers with diverse types of bacteria [6, 7]. Such diversity will ensure a healthy gut to resist pathogens. The composition of this microbiota is associated with the environment [8]. However, the core bacterial community is relatively constant across populations and geographical areas [9]. The gut community of worker honeybees is dominated by nine bacterial species clusters [10], with five core bacterial species resident mainly in hindguts [11]. The current study is aimed to characterize the gut microbial community structure of wild honeybee found in an urban area with dense settlements.

## 2. Material and Methods

### 2.1. Sample Preparation

The honeybee was captured in March 2019 from an abandoned garden in Mahakeret, Manado, North Sulawesi, Indonesia. The location is in an urban area with dense settlements. The honeybee was surface-sterilized by immersing it in 1.0% sodium hypochlorite for 10 min. Then, it is transferred into 70% ethanol for 2 min, and rinsed with sterile distilled water three times. The insect was dried using a sterile tissue paper.

### 2.2. Identification of the Honeybee

Mitochondrial DNA (mtDNA) was extracted from the base of the coxa, which is connected to the abdomen, according to Tallei et al. [12]. The mtDNA was prepared using The ZR Tissue and Insect DNA MiniPrep™ (Zymo Research) by following the instruction provided by the manufacturer. The extraction product was cleaned using The DNA Clean and Concentrator™-5 (DCC™-5) to obtain high-quality DNA for PCR. The amplification of COI (cytochrome c oxidase subunit 1) region was performed using Toyobo KOD FX Neo PCR Master Mix using primer pairs LCO1490 and HCO2198. The sequences of the primers are LCO1490 5′GGTCAACAAATCATAAAGATATTGG3′ and HCO2198 5′ TAAACTTCAGGGTGACCAAAAAATCA3' [13]. The PCR condition was as follows: 2 min initial denaturation at 95°C followed by 35 cycles of denaturation at 98°C for 10 sec, annealing at 54°C for 30 sec, elongation at 68°C for 45 min, and additional extension for 5 min at 68°C. The amplicons were sequenced using the same primer pairs at 1st BASE DNA Sequencing Services Malaysia.

### 2.3. COI Data Analysis

The chromatograms were processed following the procedure performed by Tallei and Kolondam [14]. The clean COI sequence was deposited in GenBank (http://www.ncbi.nlm.nih.gov). Identification was performed using the BLAST identity search at the same platform.

### 2.4. Bacterial gDNA Extraction and Amplification

The honeybee's gut was dissected under sterile conditions and homogenized in a FastPrep-24 Instrument at 4 m/s for 25 sec. The subsequent procedure was done according to Fatimawali et al. [15]. The extraction of gut bacterial gDNA (genomic DNA) was performed using ZymoBiomics DNA Mini Kit (Zymo Research) following the manufacturer's protocol. The gDNAs were evaluated by electrophoresis on a 0.8% agarose and analyzed using NanoDrop 1000 (Thermo Scientific, Wilmington, DE, USA). The hypervariable V3-V4 regions of 16S rRNA gene were amplified using MyTaq™ HS Red Mix (Bioline, BIO-25044) in Agilent SureCycler 8800 Thermal Cycler with the following reaction condition: initial denaturation at 95°C for 3 min, followed by 35 cycles of denaturation at 95°C for 15 sec, annealing at 52°C for 30 sec, and extension at 72°C for 45 sec, and then followed by a final extension at 72°C for 3 min.

### 2.5. 16S rRNA Library Preparation

The following procedure was done according to Tallei et al. [16]. An Illumina two-step PCR protocol was used for preparing the amplicons library. The first stage was to generate PCR products of V3-V4 regions using Nextera-style tag sequences (overhang sequences) with the following sequences: forward overhang P5-tag: 5′TCGTCGGCAGCGTCAGATGTGTAT AAGAGACAG-[locus-specific sequence] and reverse overhang P7-tag: 5′GTCTCGTGGGCTCGGAGATGTGTATAAGAGACAG-[locus-specific sequence]. The second stage used sample-specific Illumina Nextera XT dual indices (Nextera XT i7 index and Nextera XT i5 index) with the following sequences: P5-PCR index primer: 5′AATGATACGGCGACCACCGAGATCTACAC[i5]TCGTCGGCAGCGTC and P7-PCR index primer:5′CAAGCAGAAGACGGCATACGAGAT[i7]GTCTCGTGGGC TCGG. The final products were assessed using TapeStation 4200 from Agilent Technologies, HelixyteTM green dsDNA quantifying reagent, and qPCR using Jetseq library quantification Lo-Rox kit from Bioline (London, UK). The paired-end sequences were generated in a 2 × 300 bp format from MiSeq.

### 2.6. Bioinformatics Analysis

The following procedure was done according to Tallei et al. [16]. Removal of sequence adapters of the raw sequences was performed using Bbmap and merged using BBMerge (BBTools package). After alignment, trimming, and chimeras removal, all reads were clustered into OTU using UCLUST (de novo) using a 97% similarity threshold. Prior to taxonomy and diversity analysis, singleton and doubletons were removed. The samples were subsequently rarefied to the lowest number of reads among all samples.

### 2.7. Analysis of Honeybee Bacterial Diversity

The alpha diversity of the bacterial gut was calculated and analysed using PAST3 *v*. 3.24 [17]. The alpha diversity was represented by dominance (D), Simpson (1 − D), Shannon-Wiener (H′), evenness (eH/S), Margalef (Dmg), and equitability (*J*) indices [15].

## 3. Results and Discussion

### 3.1. Identification of the Honeybee

The cytochrome oxidase I (COI) sequence of the honeybee captured at the Mahakeret area has been deposited in GenBank with accession number MK880239. Based on BLAST identification, the specimen shared 99.50% identity with *Apis nigrocincta* from Sangihe Island, North Sulawesi (GenBank Accession AP018398). *Apis nigrocincta* has been recorded to inhabit Mindanao island (the Philippines), Sangihe islands (North Sulawesi, Indonesia), and the main island of Sulawesi (Indonesia). This honeybee nests in cavities near the ground, such as holes in trunks and caves [18]. Like other honeybees, this species is a generalist and visits a broad range of plants for food.

### 3.2. Bacterial Microbiome Composition of *A. nigrocincta* Gut

**Figure 1:**
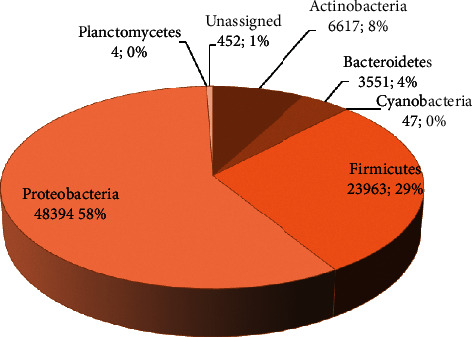
Six phyla of bacteria found in *A. nigrocincta* gut.

A large number of reads of the bacterial microbiome was produced by Illumina sequencing from the gut of *A. nigrocincta*. After the removal of chimera and singleton, there were 83,018 reads of 16S rRNA V3-V4 region sequence. In total, based on 16S rRNA sequences, the microbiome in *A. nigrocincta* gut was identified as belonging to six phyla of bacteria (from most abundant to least: Proteobacteria, Firmicutes, Actinobacteria, Bacteroidetes, Cyanobacteria, and Planctomycetes) (Figure 1). Predominant phyla included 58% Proteobacteria, 29% Firmicutes, and 8% Actinobacteria. Some previous studies demonstraing that honeybee workers have a unique microbial community that is composed predominantly of three major bacterial phyla (Firmicutes, Proteobacteria, and Actinobacteria) [10, 18–22]. Yun et al. [21] found that, on average, the insect gut bacterial microbiota was dominated by 62.1% Proteobacteria and 20.7% Firmicutes. Approximately one billion bacterial cells reside in the gut of mature honeybee workers, and ∼95% are in the hindgut [8].

### 3.3. Diversity of the Bacterial Microbiome

**Figure 2:**
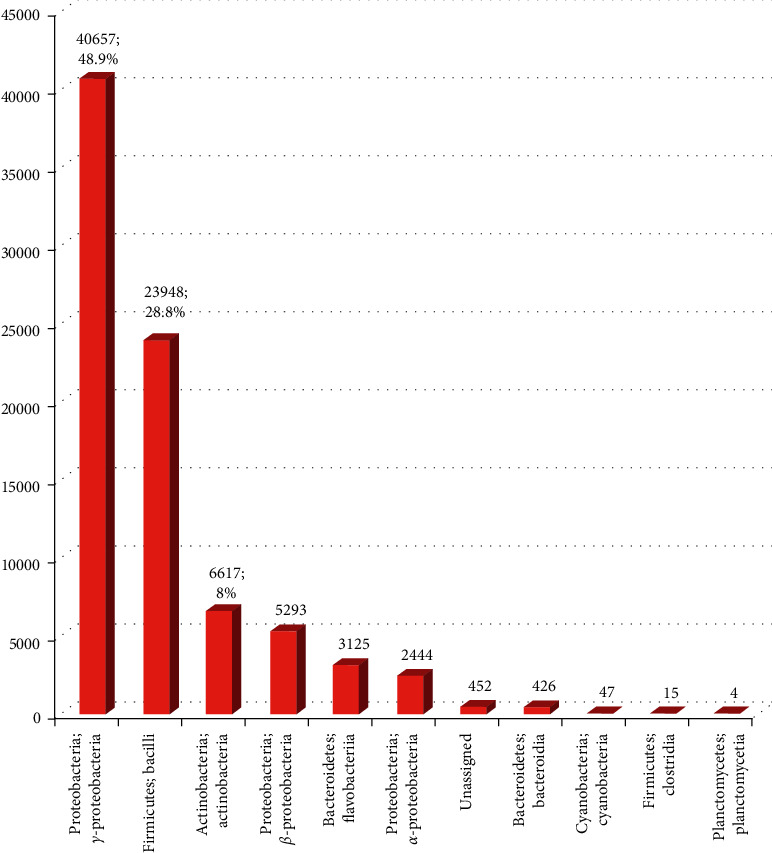
Relative frequency of bacterial class level in *A. nigrocincta* gut.

The taxonomic composition of the microbiome at the bacterial class level was ɣ-Proteobacteria (48.9%), Bacilli (28.8%), and Actinobacteria (8%) with a total of 85.7% (Figure 2). A similar finding was described by Lee et al. [23] and Horton et al. [19]. They found that the bacterial community of worker bee gut is dominated by *γ*-Proteobacteria, Bacilli, and Actinobacteria (total 90%).

**Figure 3:**
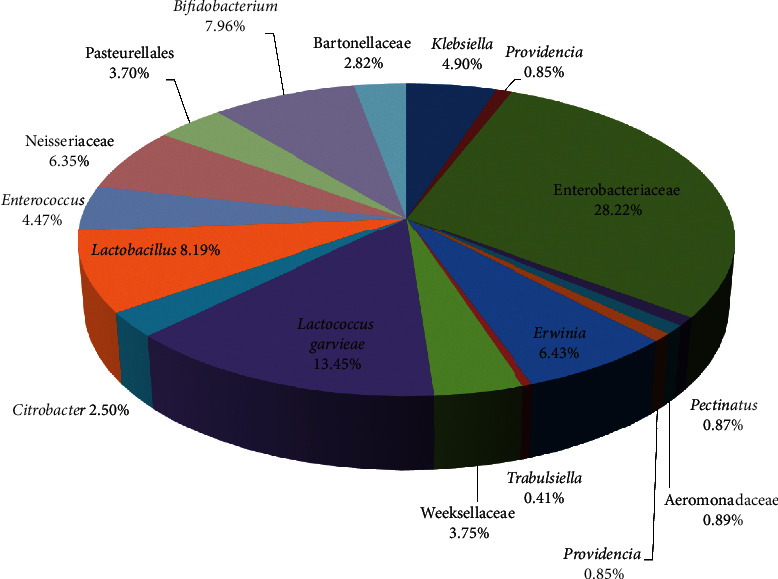
The most abundant genera (including some predominant families) in the gut of *A. nigrocincta*.

Six bacterial families were predominant: Enterobacteriaceae (44.3%), Streptococcaceae (13.2%), Lactobacillaceae (8.1%), Bifidobacteriaceae (7.9%), Neisseriaceae (6.3%), and Enterococcaceae (5.8%). The most abundant genera (Figure 3) were within Firmicutes (*Lactococcus* 13.5%; *Lactobacillus* 8.2%; *Enterococcus* 4.7%), Actinobacteria (*Bifidobacterium* 8%), and Proteobacteria (*Erwinia* 6.4%; *Klebsiella* 4.9%; *Citrobacter* 2.5%) (Figure 3). Martinson et al. [6] reported that the core clades of adult worker bee included two species from Firmicutes, one species from Actinobacteria, and six species from Proteobacteria. Throughout their lifetime, bees perform different tasks, depending on their age. This might as well contribute to the microbiome community in their guts, especially as they visit different kind of hosts.

*Enterococcus* was accounted for 4.47% of the gut bacterial population in *A. nigrocincta*. This genus is cocci-shaped lactic acid bacteria (LAB) which commonly found as gut and honeycomb microflora and known to produce an antimicrobial compound. *Enterococcus haemoperoxidus* (found in small number, 79 reads) was probably ingested by the insect from flowers. Linjordet [24] reported that this species was found in rapeseed. It can also be assumed that the insect acquired the species from water, as Svec et al. [25] reported that the bacteria were found in water. Yun et al. [26] reported that insect gut bacterial diversity was determined among others by its habitat and diet. However, the composition of the microbiome in *A. mellifera* workers is reasonably consistent. This suggests that genotypic variation does not affect the gut microbiome of the honeybee [27]. However, the previous study showed that strains in honeybees and bumblebees are host-specific, as they are only able to colonize their native hosts [28].

*Lactococcus garvieae* was found in quite a large number (11166 reads or 13.5%). Linjordet [24] found this species in willowherb (a herbaceous flowering plant in the family Onagraceae), accounting for 30% of the bacterial population. This species is a fish pathogen associated with different human infections [29, 30]. However, Zhang et al. [31] reported that *L. garviae* B301 could be potentially used as animal-feed probiotic as it improved the health of broiler chicken and enhanced their performances. They even suggested that this strain could be potentially used as a feed additive for broiler chickens. Abdelfatah and Mahboub [32] reported that *L. garviae* originating from dairy products produced a bacteriocin-like inhibitory substance. This species is considered as LAB and has been used in manufacturing cheese and fermented milk products [33].

*Morganella morganii* was only found in a small number (18 reads). The species isolated from indigenous honeybees of Saudi Arabia caused mortality of *Paenibacillus larvae* spores by 86.67% [20]. *Paenibacillus larvae* is a deadly pathogen of honeybee larvae. Lamei et al. [34] reported that there were 13 species of LAB were found in the crop of *A. mellifera* within the genera *Lactobacillus* and *Bifidobacterium*. As much as 6609 reads (7.96%) of *Bifidobacterium* and 6802 reads (8.19%) of *Lactobacillus* were detected in *A. nigrocincta* gut. These LAB could play a vital role in honeybee health, protecting them against bee pathogens [35, 36], supporting food processing such as carbohydrate metabolism [7, 23], and contributing to the antimicrobial properties of honey [37].

As reported by Huang et al. [38], the dominant genera of gut microbiota of *A. cerana*, the closest taxa of *A. nigrocincta*, were *Serratia*, *Snodgrassella*, and *Lactobacillus*. Some lactic acid bacteria such as several *Lactobacillus* species produce several antimicrobial compounds including organic acids, hydrogen peroxide, bacteriocin, reuterin, and reutericyclin. These compounds inhibit decaying and pathogenic bacteria, both Gram positive and negative, and some fungi [39, 40]. This indicates the probability that honeybees use gut bacteria as probiotics [28].

The abundance and diversity of probiotics in thehoneybee's gut are prerequisites for their health, considering that the gut is very susceptible to pathogenic and parasitic infections [34]. The gut microbial composition will show the health condition of the bees, since disruption of this composition has been associated with detrimental effects on their health [11]. Linjordet [24] reported that *L. kunkeei* and *Fructobacillus fructosus* were the most abundant in the honeybee gut. *Fructobacillus* (50 reads; 0.05%) is a fructophilic LAB that prefers fructose instead of glucose as a growth substrate. The low abundance of Bifidobacteriaceae and Lactobacillaceae was speculated to be impacted by the appearance of pathogens in the gut [41].

Genera within Enterobacteriaceae (28.22%) that were detected in *A. nigrocincta* gut, among others, were *Citrobacter* (2.0%), *Klebsiella* (4.90%), *Providencia* (0.85%), *Proteus* (0.8%), and *Erwinia* (6.43%). Seemingly, these genera are commonly found in the alimentary tract of adult bees. Their presence was dependent on neither seasonal nor food factors [42]. Disayathanoowat et al. [43] stated that *K. pneumoniae* was the most abundant species of bacteria from the midgut of the Asian honeybee (*Apis cerana indica*).

Neisseriaceae (5273 reads; 6.35%) was also found in *A. nigrocincta* gut. Liu et al. [44] found *N. meningitidis* in African honeybee (*A. mellifera*). Kwong and Moran [45] reported that this family is a gut symbiont of honeybees and bumblebees, accounting for approximately 30% of the microbiota [9]. Members of the core gut of *A. mellifera* community include *Snodgrassella alvi* (Betaproteobacteria: Neisseriales, *Gilliamella apicola,* and *Frischella perrara* (Gammaproteobacteria: Orbales) [1].

Weeksellaceae (3.75%; Bacteroidetes) was predominant in the larval and pupal stages of *Bactrocera carambolae* (Insecta: Tephritidae) [46]. Their population was higher in bees fed with bee-bread and was associated with *Nosema ceranae* infection in bees fed with sugar solution [38]. Bartonellaceae was found in a low number of reads (2.82%) in *A. nigrocincta* gut. Kešnerová et al. [48] found that *Bartonella apis* sp. nov. was a honey bee gut symbiont. *Dysgonomonas* was found in low read (0.5%). This genus has been found in other orders of insects such as Hymenoptera, Coleoptera, Lepidoptera, and Diptera, and *Drosophila* [48]. It has been shown as core members of gut microbiomes of non-Hawaiian *Drosophila* species, termites, and honeybees [49].

### 3.4. Bacterial Community Structure in the Gut of *A. nigrocincta*

Alpha diversity in terms of OTUs (lower taxa: family, genus, and species) richness and diversity was calculated using PAST3 v.3.24 (Table 1). Simpson (1-D) value 0.872 means the sample diversity is high. The index represents the probability that two individuals randomly selected from a sample will belong to different species. The dominance (*D*) value 0.128 indicated that no taxon dominated the community altogether. The value ranges from 0 to 1, and 0 represents infinite diversity [50]. Shannon index generally ranges between 1.5 and 3.5. Shannon index 2.488 showed that the diversity was moderate. The value is high as the number of OTUs increases and the distribution of individuals among the taxa becomes even. The diversity produced by Shannon-Wiener index (H′) is equivalent to one community containing equally common species of e^H^ which is termed as the effective number of species (ENS). This is the number of equally abundant species needed to produce the observed diversity values [51]. The H′ value of lower taxa of OTUs was 2.488. This has the equivalent value of diversity as a community with an effective number of species (true diversity) (e^2.488^) of 12.04 (high diversity).

**Figure 4:**
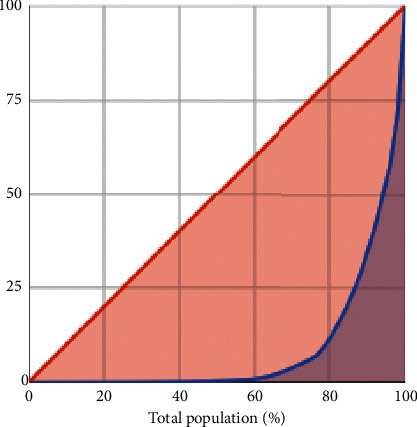
Lorenz curve of evenness of the OTUs.

Margalef index is the simplest measurement of biodiversity [52]. The value of 4.503 indicated a high level of taxa richness. The value of evenness index 0.232 indicates that there was no evenness of the community. Evenness was also calculated using a Lorenz curve. The bacteria's distribution was uneven since the curve was farther away from perfect evenness (diagonal line) (Figure 4). The more diverse the taxa, the more uneven the abundance of each taxon [52]. The composition and structure of microbial OTUs in the gut were affected by the host social status, rather than hostage [26]. Some study showed that Shannon diversity index for foraging bee is approximately 0.69 [19], 0.9 [8], 2 [53], 3 [21], and 4 [54] and for queen bumblebee after hibernation was 3 [55]. Shannon diversity index was higher for nurses, males, and queens [53]. The simpson index for foraging bees was 0.1 [21]. The evenness index for foraging bees was 0.69 [19] and 0.7 for queen bumblebee after hibernation [55].

**Figure 5:**
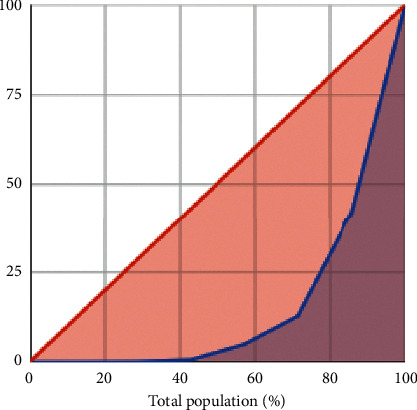
Lorenz curve of evenness of the phyla.

Alpha diversity of the phyla residing in the gut of *A. nigrocincta* was calculated using PAST3 (Table 2). The dominance (*D*) value 0.4312 indicates no phylum dominated the community completely. Simpson (1 − D) value 0.5688 means the phyla diversity was moderate. Shannon index 1.043 shows that phyla diversity was low. The H′ value of phyla was 1.043. This has the equivalent value with ENS 2.8 (slightly high). The value of evenness index 0.4053 indicates that there was a moderate evenness in the community. The value of Margalef index 4.503 indicates moderate phyla richness. Evenness was also calculated using a Lorenz curve (Figure 5). The distribution of the bacterial phyla was uneven as the curve is farther away from perfect evenness. It seems that the phylum diversity in the gut of *A. nigrocincta* as quite low. In this study, 6 phyla were found, while Yun et al. [21] found eight phyla: Firmicutes (56.65%), Proteobacteria (42.16%), Bacteroidetes (0.55%), Actinobacteria (0.41%), Cyanobacteria (0.14%), Tenericutes (0.04%), Fusobacteria (0.04%), and Acidobacteria (0.006%).

## 4. Conclusions

This present study outlines a detailed investigation of the bacterial composition and community structure in the gut of *A. nigrocincta* by high-throughput sequencing using V3-V4 16S rRNA regions. In conclusion, this study shows moderate OTUs and low phyla diversity of the bacteria. Proteobacteria predominated the phyla, Firmicutes, and Actinobacteria. This investigation of the bacterial microbiome of *A. nigrocincta* provides insight into the relationship between the gut bacterial community and the host. Hence, further studies will be required to elucidate more about the relationship between bacterial symbionts and the insect. Furthermore, this finding can be used as a basis for bioprospecting research.

## Figures and Tables

**Table 1:** Alpha diversity of the OTUs in *A. nigrocincta* gut calculated using PAST3.

Alpha diversity	Value	Interpretation
Dominance D	0.128 ± 0.001	No dominance
Simpson D' = 1 − D	0.872 ± 0.001	High diversity
Shannon H′	2.488 ± 0.008	Moderate diversity
Evenness e^H/S^	0.232 ± 0.002	No evenness
Margalef (Dmg)	4.03 ± 0.000	High richness
Equitability J	0.630 ± 0.002	

**Table 2:** Alpha diversity of the phyla in *A. nigrocincta* gut calculated using PAST3.

Alpha diversity	Value	Interpretation
Dominance D	0.431 ± 0.003	Moderate dominance
Simpson 1 − D	0.569 ± 0.003	Moderate diversity
Shannon H	1.043 ± 0.006	Low diversity
Evenness e^H/S^	0.405 ± 0.002	Moderate evenness
Margalef (Dmg)	0.530 ± 0.000	Moderate richness
Equitability J	0.536 ± 0.003	

## Data Availability

The data used to support the findings of this study are available from the corresponding author upon request.
